# The Pipeline Repertoire for Ig-Seq Analysis

**DOI:** 10.3389/fimmu.2019.00899

**Published:** 2019-04-30

**Authors:** Laura López-Santibáñez-Jácome, S. Eréndira Avendaño-Vázquez, Carlos Fabián Flores-Jasso

**Affiliations:** ^1^Consorcio de Metabolismo de RNA, Instituto Nacional de Medicina Genómica, Mexico City, Mexico; ^2^Maestría en Ciencia de Datos, Instituto Tecnológico Autónomo de México, Mexico City, Mexico

**Keywords:** Ig-Seq, antibody repertoire, pipeline, V(D)J alignment, pre-processing

## Abstract

With the advent of high-throughput sequencing of immunoglobulin genes (Ig-Seq), the understanding of antibody repertoires and their dynamics among individuals and populations has become an exciting area of research. There is an increasing number of computational tools that aid in every step of the immune repertoire characterization. However, since not all tools function identically, every pipeline has its unique rationale and capabilities, creating a rich blend of useful features that may appear intimidating for newcomer laboratories with the desire to plunge into immune repertoire analysis to expand and improve their research; hence, all pipeline strengths and differences may not seem evident. In this review we provide a practical and organized list of the current set of computational tools, focusing on their most attractive features and differences in order to carry out the characterization of antibody repertoires so that the reader better decides a strategic approach for the experimental design, and computational pathways for the analyses of immune repertoires.

## Background

The study of antibody repertoires by high-throughput sequencing prompted many groups to develop computational pipelines that aid in the processing of large amounts of sequencing data in order to categorize and understand the diversity and dynamics of repertoires in individuals (1, 2). Practically, every maturation step can be followed experimentally by high-throughput sequencing, giving us the opportunity to analyze how the diverse exposure to antigens has a distinctive effect on a myriad of individual B cells, either at transcriptomic, or genomic level (3–5). As new discoveries arise in the immunology field, novel computational tools have emerged to adapt their algorithms to provide more accurate and statistically robust analyses (6, 7). Likewise, computational pipelines have also helped to unveil details previously unknown about the antibody repertoire; exhibiting the intertwined relationship that exists between modern antibody repertoire analysis and computational immunology (4, 8–15). Since the study of antibody repertoires can be addressed from many biological aspects, there is a concomitant diverse set of computational algorithms tailored to many purposes. Whereas, high-throughput sequencing has become more available for most laboratories, there is a lag in the expertise required to plunge into the current computational pipelines developed for immunoglobulin sequencing (or Ig-Seq). With the large amount of software devoted for a specific (or all) processing step(s), the analysis of antibody repertoires may seem intimidating for newcomer laboratories; as the necessary processing steps to fulfill a specific type of analysis, or the reason for using a specific tool may not be as evident.

In this review, we focus on the current repertoire of some of the most widely used computational pipelines for Ig-Seq and provide a comparison of all the specific processes they perform. We begin by briefly explaining the pre-processing step and also the measurable features of antibody repertoires and their basic rationale, to then describe the strengths and differences of each pipeline, emphasizing where the computational pipelines may converge and diverge to explore the repertoire biogenesis process. The measurable features associated with the antibody repertoire discussed in this review are: V(D)J germline assignment, clonal grouping, mutation analysis, evolution and convergence of antibody repertoires. We selected the computational tools discussed in this review based on their relevance (i.e., attention or citations received since their publication), continuous maintenance (at least one in the last 5 years), and up to date with the current sequencing platforms available. We also provide a table for all the pipelines discussed that displays their differences, and it could serve as utilitarian guide and reference for Ig-Seq analysis, or project design. Based on our analysis, we organized the table with the current pipeline repertoire into three main groups defined by the type and variety of analyses each performs: Broad spectrum, Modular and Specialized pipelines. The table is portrayed so that its printouts can be revisited frequently, and the pipeline's similarities and differences are spotted easily.

While this review focuses only on the pipelines for antibody and B cell repertoire, many pipelines manage T-cell receptors (TCRs) as well. For analysis of TCR repertoire analysis, sample and library preparation, or the mathematical basis for the statistical rationale employed by some of the pipelines discussed here, the reader may refer to other thorough reviews published recently (16–21).

## Pre-processing

The goal of the data pre-processing step is to transform Ig-Seq raw reads into error corrected sequences. Although results are not significantly different between methodologies, the pre-processing steps may vary depending on the amplification and sequencing methods employed (21–23). Due to the large extent of variability that gives rise to all B-cell clones, the identification of antibody repertoires and their germline assignment is intricate and largely compromised by biases and errors introduced during library preparation and sequencing; unlike the computational analysis commonly done for other types of high-throughput sequencing of nucleic acids (1). Because B cells undergo V(D)J recombination and Somatic Hypermutation (*SHM*, Boxes 1, 2), the sequences of interest can only be mapped to the reference genome partially. Therefore, errors introduced during the library preparation can be falsely identified as part of the true sequence of the antibody. The first approach to identify and minimize errors introduced during the library preparation was the establishment of Unique Molecular Identifiers (UMI) before the PCR amplification (19, 20, 27). This approach consists of introducing oligomers during the retro-transcription with the purpose to identify each input molecule; this allows distinguishing whether polymerization errors were introduced early in the PCR, or whether they rather reflect a biological change in a given sequence. Along with UMI, the accuracy of the repertoire reproduction can also be improved significantly by implementing molecular amplification fingerprinting (MAF, see Glossary of Terms), which uses UMI tagging before and during multiplex PCR amplification (28). Importantly, the use and identity of UMI and MAFs should be decided before library preparation because further processing steps require to specify if they were used or not.

Box 1 **V(D)J recombination**

Antibodies are produced by a developmentally ordered series of somatic gene rearrangement events that occur exclusively in developing lymphocytes. Antibodies consist of heavy (μ, alfa, δ, γ, ε) and light chains (κ, λ), each of which contains a variable and a constant domain. Antigen binding occurs in the variable domain, which is comprised of one variable (V), one diversity (D) and one joining (J) gene segment in heavy chains and one variable (V) and one joining (J) segment in light chains. The germline V(D)J genes contain approximately 41 different V segments, 23 different D segments and 6 different J segments (24, 25). However, during B cell maturation —more specifically, when the precursor B cell matures into a naive B cell— the segments are reduced to only one V, one D and one J gene segment. This process is called somatic recombination (VDJ recombination) and provides combinatorial diversity to the antibodies (26). Furthermore, the recombination process often results in non-templated mutations like the addition or deletion of nucleotides at the junctions between ligated gene segments. The site of the V(D)J gene segment ligation, also known as the complementarity-determining-region 3 (CDR-H3), is the most diverse component in terms of length and sequence of the antibody heavy chain (1).

Box 2 **Somatic Hypermutation (SHM)**

When the immune system encounters an antigen for the first time, T-cell helpers will stimulate naive B cells to mature into antigen specific B cells. The maturation process consists of a B cell clonal expansion followed by somatic hypermutation. During SHM, activation-induced cytidine deaminase (AID) introduces non-templated mutations into the variable region of the antibody genes (1). AID also mediates class switch recombination, which generates antibodies bearing different constant regions (24). B cells expressing somatically mutated, high-antigen-affinity BCRs undergo preferential expansion and survival, a processed referred to as *affinity maturation*. As a result, B cells bearing the highest-affinity antibodies differentiate into plasma cells, or long-lived memory B cells capable of mediating rapid recall responses to the same antigen.

Both, V(D)J recombination and SHM, introduce non-templated mutations into the immunoglobulin genes, which make the Ig-Seq data pre-process and analysis intricate. Once the Ig-Seq is performed, the resulted reads will not align perfectly to the germline and the misalignments could be due to either the natural maturation process of the antibodies or to PCR and sequencing errors.

The typical steps of data pre-processing are:

***Quality control and read annotation***. Since BCR sequences could differ theoretically from one another by a single nucleotide, keeping high quality reads is of utmost importance (29). The output obtained from NGS is a FASTQ file that contains each read sequenced and information about its quality per base; referred to as *Phred quality score*. Regularly, reads with a Phred score ≥20 are considered acceptable, whereas reads below 20 are discarded. After quality score analysis, the information introduced to each sample during library preparation must be annotated and masked or removed for each read —for example, annotation of average quality and UMI/adaptors used.
***Building consensus sequences***. The goal of this step is to cluster all reads by UMI and to build a consensus sequence that has minimal amplification errors. Each UMI cluster results in a single consensus sequence with the most reliable base calls. Using UMI ensures that all the reads coming from the same mRNA molecule will have the same oligomer sequence introduced during the retro-transcription. The amplification of errors and biases are then corrected by clustering the UMI.
***Assembly of paired-end reads***. When performing paired-end sequencing, the reads must be assembled into one read. In paired-end sequencing, the nucleic acid fragment sizes are selected so that the sequences can be read from both ends (5′ and 3′), and overlap with each other to some extent. Assembling the two cognate reads into a single sequence can be done by scoring different possible overlaps and by selecting the most statistically significant.

## V(D)J Germline Alignment

The V(D)J germline assignment is one of the most important steps in the processing of Ig-Seq data. The goal of this step is to infer the correct germline alleles that recombined to produce each BCR/antibody. A good germline inference is critical to identify correctly somatic mutations for each read, to cluster into clonal groups, and to have a fair diversity approximation (30). Most commonly, this inference is done by applying an algorithm to choose the best match among a set of potential germline segments from a database of known segment alleles.

### Assessing Germline Alignment of New Alleles

Currently, most germline alignment and assignment tools compare the reads to existing databases of known alleles. Since all existing databases are incomplete, should novel polymorphisms appear in a sequencing study of Ig repertoire, these are difficult to differentiate from the frequent occurrence of somatic hypermutations in antibody sequences (31). Thus, the alignment of reads to only known genes may yield inaccurate results for sequences with previously undiscovered alleles —the read will be aligned to the closest germline gene, and the new allele variance will be incorrectly identified as a result of somatic hypermutation (23, 32, 33). To address this limitation, a distinct collection of tools has been developed to identify novel alleles, aimed to generate personalized germline databases containing the specific sets of alleles carried by individuals (23, 31, 34–38).

## Clonal Grouping/Clonotyping

The goal of this stage is to group antibodies/BCRs to facilitate lineage reconstruction and diversity analysis. In clonotype grouping, lineage trees are often constructed since they offer a visual display of the relationships among antibodies, and may also be helpful to understand temporal aspects of affinity maturation. There are alternative definitions for clonotypes that govern the type of analysis executed depending on the grouping criteria; one widely used, for example, is by grouping all clonotypes that descend from the same naive B cell but differ only because of their SHM process (13). Clonal grouping, under this example, is defined at the nucleotide level (due to somatic hypermutation occurs on the DNA): reads with the same V and J alleles and a threshold nucleotide difference at the junction region (CDR3) are grouped to form a clonotype. Some pipelines define clonotypes differently, for example, those that consider each clone as a unique antibody; or those that group them by amino acid sequences, which considers every read in the group reacts to the same antigen (23, 30–38). A strategy aimed to provide a more robust clonotyping process, independent of any definition, is single linkage clustering (a statistical method for hierarchical clustering), which defines the distance between groups as the minimum distance between all pairs of points from the given groups (39, 40).

## Repertoire Characterization and Analysis

### Diversity

Antibody/BCR diversity is associated with an effective immune response against pathogen agents (41). Despite the maximum theoretical amino acid diversity of ~10^14^ antibodies/BCRs, the effective repertoire is attributable to a set only ~10^11^ in humans due to the V, D, and J genes (1). This enormous diversity has led to the development of an increasing number of computational tools designed to tackle the concomitant complexity of immune repertoires. Typically, two complementary modules constitute diversity analyses:

#### Diversity Quantification and Analysis of the Sequenced Sample

Diversity quantification refers to a basic characterization and statistics of the repertoire that may include some or all of the following: mean clonotype sizes and their read counts, number of non-functional clonotypes, CDR3 region characterization, identification of the most used V, D and J alleles in the repertoire, and the most frequent VJ combinations. The latter, can be visualized either through heatmaps, histograms or pie charts. Furthermore, if a 3′ primer was set to cover the C region during the library construction, it is possible to perform an analysis to identify the most abundant isotype; this is especially relevant when conducting protocols for “before-and-after” vaccination and their respective repertoire analysis (3, 9, 12, 14, 42–44). The statistical quantification and comparison of clonotype diversity is primarily calculated using the *generalized diversity index* —a general mathematical formulation commonly used in ecology to assess diversity, developed by Hill in 1973 (45).

Other indices used for repertoire diversity quantification that derive from Hill's capture a different clonal subset of the clonal frequency distribution (2). These include the species richness index, the Shannon Weiner index, the inverse Simpson index, the Berger Parker index (also referred to as the *reciprocal abundance of the largest clone*) the Gini index, and Chao1 index (46, 47).

#### Estimation of Total Diversity

It is estimated that from the total number of theoretically possible B cell clones in an individual, ~28% is present in lymph nodes, ~23% in spleen, ~19% in intestinal mucosa, ~17% in bone marrow, and only ~2% in peripheral blood; in practical terms, these percentages account for the ~10^11^ BCR/antibody clones that are the product of the adaptive immune response (48). With the current sequencing technology, the maximum number of reads a platform can achieve in a single run is 2 × 10^9^ —few orders of magnitude below the CDRs repertoire attributable to recombination and SHM. This implies that only a fraction of the total diversity repertoire can be identified by Ig-Seq. Therefore, the total diversity analysis must include the estimation of the undetected clones —mostly done using a rarefaction-based method (2). Rarefaction is a type of analysis (also commonly employed in ecology) that allows the calculation of the total species richness, and it is commonly portrayed as a “rarefaction curve” that plots the expected number of species as a function of the number of samples (49, 50). Species (or in this case, read counts) are averaged over multiple resamples of the data to obtain the expected number of species as a function of the number of individuals (51). All the computational tools discussed here that support the estimation of total diversity by using a rarefaction-based method.

### Mutation Analysis

High affinity BCRs/antibodies are the product of mutational events that accumulate during B cell maturation. The purpose of mutation analysis is to gain insight of the maturation process that B cells underwent during the course of an immune response and their encounter with antigens/foreign epitopes (4, 29, 52). As mutations accumulate in the CDRs, it is possible to identify hotspots that may give rise to a certain clonal population, providing even more understanding of the lineage and evolution process that took place at specific time points, or immunological events. The common features that build a mutation analysis enclose: mutation frequencies, mutations by position and hotspots identification, mutation types (i.e., number of synonymous and non-synonymous mutations which may indicate potential lineages under antigen-driven selection) and selection pressure —selection pressure is calculated by comparing the observed frequency of non-synonymous mutations and the expected number which takes into account hot- and cold-spots, and nucleotide substitution bias (53). An increased frequency of replacements indicates positive selection whereas decreased frequency indicates negative selection —CDRs are expected to be under positive selection, whereas framework regions under negative selection.

### Evolution of Repertoire/Clonal Dynamics

Immune repertoires are highly dynamic (42, 44). When the immune system encounters an antigen, memory cells may be activated to produce already existent antibodies or naive B cells may hypermutate the variable regions of the antibody thus forming a B cell lineage with new plasma cells producing new antibodies (3). Analyzing how the antibody repertoire evolves throughout time provides an insight into how pathogens, vaccines, and even self-epitopes shape our humoral response (3, 9, 12, 14, 42–44, 54–56). Although the evolution of repertoires can be studied at one single time point with phylogenetic trees that portray clonal dynamics, multiple time points help to generate more accurate and robust ontogenic analysis and these can be portrayed as stream graphs, longitudinal phylogenetic “birthday” trees, stack-plots or clonotype tracking heatmaps.

In order to infer the antigen driven evolution of antibody repertoires, a phylogenetic method [such as neighbor joining (NJ), maximum parsimony (MP), maximum likelihood (ML), or Bayesian inference] is applied to the set of Ig reads (5, 57), which results in the compartmentalization of all sequence reads into clades. Each clade contains all the sequences that share a common ancestor (i.e., that derive from the same naive B-cell). The phylogenetic tree is constructed using the resulting clades. Although the phylogenetic methods currently used are a fair approximation, most rely on assumptions that may be true for species evolution, but might be invalid for antigen driven evolution of antibodies; this inexorably would decrease the accuracy of the clade prediction.

In order to track clonotypes throughout multiple time points, a comparison between the repertoires at the different time points is performed (5, 35, 58). This comparison allows the identification of antibodies that are present in more than one time point.

### Repertoire Convergence

Convergence analysis refers to a phenomenon that occurs when identical (or highly similar) immune receptor sequences are shared by two or more individuals in the context of infection or vaccination, and provides evidence that some antigenic stimuli can provoke relatively predictable responses, which is expected to occur in genetically similar populations (10, 12, 43, 59). For example, it is been shown that the similarity of gene segments in productive IgH repertoires of twin brothers is greater in non-mutated than mutated Ig genes (12). This suggests that the process governing naive B-cell repertoire generation is more similar in related individuals, but that the subsequent antigen-driven evolution might be less genetically controlled. However, it is also conceivable that heritable biases in the naïve repertoire may affect the likelihood of clones with specific recombination becoming activated and transiting to the memory compartment (15). Repertoire convergence analysis may be of substantial importance for the prediction and manipulation of adaptive immunity as well as vaccination and gene therapy.

One way to determine the level of repertoire convergence is by finding the clonotype overlap across individuals (either at nucleotide or amino acid level), and express it as a percentage —normalized by the clonal size of the samples used (2). Hence, an accurate clonal clustering is of utmost importance in providing robustness to convergence studies. Here, the definition of clonotype employed usually refers to single sequences, and therefore two samples containing the same clonotype have a convergent sequence. In the case when clonotypes are treated not as single, but clusters of sequences, two samples will share a cluster if at least one sequence is shared between them. In convergence studies, it is common to use indices that provide additional information by integrating the clonal frequency of the compared clones; for example the Morisita-Horn index (2). Another index, the Repertoire Dissimilarity Index (RDI), enables the quantification of the average variation among repertoires (60). The RDI is a non-parametric method for directly comparing repertoires, with the goal of rigorously quantifying differences in V, D, and J gene segment utilization. Visualization of repertoire convergence can be achieved through Venn Diagrams, abundance plots, overlap circos-plots, or hierarchical clustering dendrograms. For a more thorough overview of the pre-processing steps and types of analysis of Ig-Seq refer to Miho et al. (2), Yaari and Kleinstein (23), and Galson et al. (44).

## The Pipeline Repertoire

The identification of adaptive immune response receptors has yielded a number of diverse pipelines that perform part, or all the segments discussed above among others specifically tailored to cover specific research needs and questions. As new discoveries arise in the immunology field, new tools are generated to manage the concomitant changes and implications in the antibody repertoire, and *vice versa*. This section compiles the current, most widely used pipelines, and a brief description of the key features they offer to allow research at the cutting-edge of antibody repertoire analysis. The full list of features and capabilities is summarized in Supplementary Table 1. The table is portrayed so that its printouts can be revisited frequently to easily spot how each computational tool converges with —and also differs from— one another, which in turn help to gauge the reach of the experimental set up.

Based on our analysis and comparison, we categorized the tools according to their capabilities. The pipelines that can handle most of the computational analyses discussed here are referred to as *Broad Spectrum* Pipelines. This comprises IgReC, ImmunediveRsity, the Immcantation Framework, IGGalaxy, and VDJServer. Computational tools that perform V(D)J Alignment and Clonal Grouping analyses, either as stand-alone or by wrapping other tools, are referred to as *Modular* Pipelines. Applications that do not perform V(D)J Alignment, and rather focus on specific computational steps of repertoire characterization (such as clonal dynamics, evolution, and convergence) are referred to as *Specialized* Pipelines. Lastly, the tools that only perform V(D)J alignment are grouped under the *V(D)J Alignment* category. The combination of the Broad Spectrum, Modular, Specialized and V(D)J Alignment pipelines allows constructing an interrelated analysis tailored for each specific need, and facilitates the study of the fairly complex immune response.

### Broad Spectrum Pipelines

#### IgReC

##### Pre-processing

*IgReC* is part of the Y-tools framework. It is an algorithm for constructing antibody repertoires from high-throughput sequencing datasets. It takes as an input both, single and paired-end reads (31), and provides the option to correct errors using UMI. In cases where no UMI were added to the libraries, the error correction is performed by clustering the reads by using the Hamming graph —whose vertices represent unique reads that are then used to build a consensus sequence.

##### V(D)J Germline Assignment

IgReC aligns all reads to the database of Ig germline genes and then discards the unaligned ones (31). To improve its efficiency, IgReC labels the V and J segments based on a fast algorithm for finding the longest subsequence of k-mers between reads and germline segments; the remaining reads are then realigned. This process bypasses the time consuming computation of extended Hamming distances.

##### Clonal Grouping/Clonotyping

IgReC tackles clonotyping by building a Hamming graph to identify similar reads to then identify dense sub-graphs that become a clonotype of highly related antibodies. The visualization is done through a lineage tree or hamming graphics.

##### Diversity and Mutation Analysis

These steps are performed by IgDiversityAnalyzer, a complementary tool for annotation, diversity analysis and mutational analysis of full-length adaptive immune repertoires (31). The tool is capable of creating summary tables with pertinent information as well as plots for visualization.

#### ImmunediveRsity

##### Pre-processing

ImmunediveRsity is a tool primarily based in R programming for the integral analysis of B cell repertoire data. Although it is similar to other contemporary developed tools like MiGEC, MiXCR, and pRESTO, it was the first to offer a beginning to end analysis, including ready to publish plots, within the same tool (39). ImmunediveRsity supports both single-, and paired-end formats, and was originally designed for libraries prepared by RACE amplification. For the pre-processing of data, it performs the quality filtering and designed imm-illumina, a tool for the paired-end read assembly. One important feature to note is that ImmunediveRsity does not perform amplification correction based on UMI itself; instead, it calls *Acacia*, an error-correction tool, after the V(D)J alignment.

##### V(D)J Germline Assignment

To assign the V(D) J segments, ImmunediveRsity uses IgBLAST to align each read to the current IMGT database. Moreover, the pipeline provides a file with the CDR3 for each read.

##### Clonal Grouping/Clonotyping

In ImmunediveRsity the clonotype is defined by a group of identical reads; therefore each clonotype is a unique antibody. ImmunediveRsity uses the clonotyping to correct library preparation errors. Additionally, the pipeline provides the clonal abundance and a visual representation of the clonotype lineages. Instead of a lineage tree, the group visualization is a graphical network of the clonotype and their lineages; which could be helpful for a population dynamics approach.

##### Diversity

The ImmunediveRsity pipeline performs CDR3 identification and characterization, VJ usage heatmaps, diversity quantification (capable of performing Shannon Weiner index, Shannon Weiner normalized or weighted index and the Gini coefficient) and can plot the rarefaction curves for total diversity estimation.

##### Mutation Analysis

It reports the synonymous and non-synonymous mutations.

#### Immcantation Framework

##### Pre-processing

The Immcantation Framework includes the tool pRESTO which is capable of performing all the stages of pre-processing, from raw sequences up until the paired-end assembly if required. In order to fulfill all of its capacities, pRESTO is composed of a set of stand-alone tools that can be combined to construct commands specific to individual protocols (61). It supports multiplexed and RACE samples and is also capable of performing de-multiplexing if this was not done by the sequencing facility. pRESTO supports single-end sequencing, and it can assemble reads that may or may not overlap for the paired-end format. Reads processing can be carried out with or without UMI, making it a very flexible, adaptable and suitable tool for many existing protocols.

##### V(D)J Germline and New Allele Assignment

The Tool for the Immunoglobulin genotype Elucidation (TIgER), identifies novel VJ segment alleles, and constructs a personalized germline database (32). This information is then used to improve the initial V segment assignments from existing tools, like IMGT/HighV-QUEST (62, 63). This means that the alignment must be performed beforehand and then TIgER will correct the misinterpreted new alleles and create a personalized germline database. The required input for this tool is a germline database in IMGT-gapped fasta format, and a table of reads with the preliminary V and J alleles, and length junction. The table can easily be created with the output of Change-O (also part of the Immcantation portal), or IMGT, V-QUEST and IgBLAST (62–69).

##### Clonal Grouping/Clonotyping

For this step, the portal is assisted by the package Change-O for standardizing the output of alignment software such as IMGT HIGH V-QUEST or IgBLAST, clonal grouping and germline reconstruction (64). Change-O allows processing of reads that contain a premature stop codon, and would be non-functional. It groups clonotypes by V and J allele and the nucleotide Hamming distance; it also provides the option to choose which substitution model to use for calculating distance between sequences. The different substitution models will lead to the different definitions of clonotype. Available models are: nucleotide Hamming distance, amino acid Hamming distance, human specific single nucleotide model and 5-mer content model. It also enables to choose between single, average or complete linkage for the type of hierarchical clustering. The Immcantation framework also includes Alakazam, which is an R package that serves as interface for interacting with the output of Change-O and pRESTO. Alakazam takes the clonotypes grouped by Change-O and plots the lineage tree of the repertoire.

##### Diversity

This step is also performed by Alakazam, which performs basic repertoire characterization, diversity quantification and total diversity quantification (64). The tool calculates V(D)J allele, gene or family usage, as well as physic-chemical properties of the amino acid sequences. For diversity quantification, the species richness, the Shannon Weiner index, the inverse Simpson index and the Berger Parker Index, can be calculated. When inferring the complete clonal abundance, Alakazam uses the Chao estimation as approximation for the number of seen clones, and then applies the relative abundance correction and unseen clone frequencies described by Chao et al. (11, 24, 46, 49, 64). Furthermore, the tool provides the rarefaction curve.

##### Mutation Analysis

Its uses the R package SHazaM to quantify the mutational load and it includes tools to build the SHM targeting models from the data. Moreover, the package includes a tool to analyze the selection pressure (64).

##### Convergence

The RDI package is part of the Immcantation analysis framework and provides methods for visualizing and calculating the Repertoire Dissimilarity Index (51).

#### IGGalaxy

IGGalaxy is a web-based application that uses Galaxy's graphical user interface and it can be used on an individual computer and on a server (25). The application provides an *Experimental Design* tool that allows samples to be merged with an experimental design structure (i.e., name samples and replicates).

##### Pre-processing

FASTA reads must be preprocessed prior to analysis; this can be performed either by existing tools in Galaxy and IGGalaxy, or with another tool before uploading the data into the application.

##### V(D)J Germline Assignment

The germline alignment can be performed using either the IgBlast wrapper, or the IMGT HighV-QUEST wrapper provided by IGGalaxy.

##### Clonal Grouping/Clonotyping

IGGalaxy provides clusters the definition by the given definition of unique sequence (either VJ CDR-AA or VJ CDR nucleotide).

##### Diversity

The *Report* tool summarizes the frequency of V, D, and J chains as bar charts, as well as the combination V-D, V-J and D-J heatmaps based on the definition of unique sequence. IGGalaxy *Report* tool provides further analysis using existing Galaxy genomic and statistical analysis functionality.

#### VDJServer

VDJServer is the first cloud-based analysis portal for immune repertoire sequence data that provides access to a number of tools for a start-to-finish analysis workflow (70). The portal is accessible through a standard web browser via a user-friendly graphical user interface, which facilitates its use by research groups that lack some bioinformatics expertise. Moreover, VDJServer provides free access to High Performance Computing (HPC) at the Texas Advanced Computing Center.

##### Pre-processing

The portal provides access to pRESTO or VDJPipe (71). VDJServer automatically calculates base composition statistics and read quality statistics before and after pre-processing and provides comparative visualization for user assessment.

##### V(D)J Germline and New Allele Assignment

VDJServer employs IgBlast to perform the alignment against the IMGT database.

##### Clonal Grouping/Clonotyping

The portal uses either Change-O or RepSum for clonal grouping and annotation (70).

##### Diversity

The portal provides access to RepCalc and Alakazam (64).

##### Mutation Analysis

The portal provides access to SHazaM.

### Modular Pipelines

#### MiGEC

##### Pre-processing

MiGEC was introduced as one of the first pipelines that processed UMI-tagged reads. It performs all pre-processing steps, including the de-multiplexing, but differs on the specific order mentioned above (7, 20). For example, assuming that de-multiplexing is done, the first step in MiGEC is the clustering by UMI, creating molecular identifier groups accompanied by the size distribution and statistics for each group. Once the groups are created, the average quality score of the whole group is calculated. Low quality groups are discarded as they are assumed to contain errors at the early stages of PCR. Furthermore, MiGEC performs two stages for error correcting and the building of the consensus sequence. The first correction step identifies the dominant sequence variant and corrects minor sequence variants within each group. The second step builds the consensus sequence and eliminates the variants produced by hotspot PCR errors. This two steps correction process minimizes error introduced by amplification and eliminates almost all artificial diversity (7). In terms of amplification, MiGEC supports both multiplexing and RACE amplification. It only supports paired-end sequencing with UMI.

##### V(D)J Assignment

MiGEC is capable of mapping the V, D and J segment as well as the extraction of the CDR3 region by using the IMGT database.

##### Clonal Grouping/Clonotyping

In MiGEC each consensus sequence assembled is a clonotype after the amplification and sequencing correction with UMI, which implies that every sequence is a unique antibody/BCR. Each clonotype is specified by count, fraction, V, D, and J segment identifier list and CDR3 nucleotide and amino acid sequence.

#### IMSEQ

##### Pre-processing

IMSEQ is a tool that derives clonotype repertoires from NGS data and introduces a new routine for handling errors produced during the library preparation (40). It supports both single and paired-end formats and it performs all steps of the data pre-processing stage. The quality filtering process allows the filtering by a Phred-like score, or by “clustered” Phred-like score. Furthermore, IMSEQ allows for an amplification error correction without the need of UMI —step done by adding a second quality-filtering step after clonal grouping. Since errors introduced during the amplification process produce new clonotypes that are highly similar to the true clonotype, IMSEQ checks for every identified clonotype cluster whether it is likely or not to be erroneously derived from another clonotype cluster at the post-processing error correction step. In the case that it is indeed likely to be derived from another clonotype cluster, the clonotype cluster is attributed to an amplification error and therefore eliminated (40).

##### V(D)J Assignment

To identify efficiently the V and J reference genes that yield the best scoring overlap alignments against each read, IMSEQ initially matches a set of short segment substrings, denoted as segment core fragments (SCFs), against each read. After the VJ assignment, the CDR3 region is determined.

##### Clonal Grouping/Clonotyping

For IMSEQ each clone is a unique antibody. Additionally, when CDR3 reads are out of frame, or contain a stop codon inside its region, the read is rejected and considered non-functional. An interesting feature of IMSEQ is that the clustering is also used to provide a framework for PCR and sequencing error correction without the need for UMI [see V(D)J germline alignment above].

#### MiXCR

##### Pre-processing

*MiXCR* is a very simple, yet flexible tool that handles paired- and single-end reads, supports both partial (only variable region) and full-length (full heavy chain) profiling, considers sequence quality and corrects PCR and sequencing errors (72). Since MiXCR does not support libraries prepared with UMI, the error correction step is done by assembling the clonotypes with a heuristic multi-layer clustering that can work with and without UMI. Furthermore, MiXCR supports RACE amplification and RNA-Seq methods.

##### V(D)J Germline Assignment

*MiXCR* employs built-in library of reference V, D, J, and C gene sequences based on corresponding loci from the GenBank database. The pipeline also offers the option to use external libraries such as IMGT.

##### Clonal Grouping/Clonotyping

MiXCR groups clonotypes by their CDR3s by default, and therefore assembles clones by unique antibody/BCR sequences —a feature that could be modified if desired. A key aspect of MiXCR is that it offers capability to choose the gene regions (V, D, J, CDR3, and C) to be used for the assembling of the clonotypes.

#### LymAnalyzer

LymAnalyzer is a software that receives FASTAQ files and starts its processing at the alignment of the V(D)J genes to the reference alleles. It provides both command line and GUI versions (73).

##### V(D)J Germline Assignment

The software uses an alignment algorithm based on fast-tag-searching to map the input sequence to the reference V and J segments, and uses the Hamming distance to choose the for best match (74). Since is shorter than VJ, the alignment of the D segment is done by removing the V and J regions before applying the alignment algorithm. LymAnalyzer has better performance in its accuracy compared to MiXCR. By using the default settings, all reference genes are derived from the most recent update of IMGT database. LymAnalyzer also offers the option to import your own reference database and perform the CDR3 extraction.

##### Clonal Grouping/Clonotyping

The sequences are grouped when they contain the same V(D)J gene and have identical CDR3 nucleotide sequence.

##### Mutation Analysis

LymAnalyzer generates the mutation trees using a method that aims to reveal the minimal steps that could have led to the observed sequence. Furthermore, the tool provides the hypermutation tree for Ig-Seq data.

#### Partis

##### V(D)J Germline Assignment

Partis is a fast, flexible, and open source framework based on the Hidden Markov Model (HMM) to analyze BCR sequences (30). By using the HMM *factorization* strategy, Partis performs V(D)J alignment using a database reference of choice. The germline reference database must be downloaded separately.

##### Clonal Grouping/Clonotyping

The framework clusters the sequences by lineage based on a multi-hidden Markov Model (30, 75).

##### Mutation Analysis

Partis reports mutation frequencies as well as of nucleotides corresponding to the non-templated insertions between the V and D segments and D and J segments.

#### ImmuneDB

##### V(D)J Germline Assignment

ImmuneDB is a system for analyzing vast amounts of heavy chain variable region sequences and exploring the resulting data (76). It uses MySQL as a database and accepts pre-annotated sequences in Change-O format as input. ImmuneDB implements a gene anchoring method for V and J identification. This package requires that V and J germlines be downloaded separately and specified in two separate FASTA files; each must comply with the IMGT formats. The tool assigns each sequence a V and J gene, but it also calculates statistics such as how well the sequence matches the germline, whether there is a probable insertion or deletion, and the extension of the V and J.

##### Clonal Grouping/Clonotyping

ImmuneDB clusters the reads based on CDR-3 amino acid similarity. Therefore, all the reads in one group react against the same antigen. It provides the clonal lineage tree for visualization of the grouping.

##### Diversity

The tool performs V and CDR length distribution as well as V and J gene usage.

#### Vidjil

Vidjil is an open source platform for the inspection, analysis and the tracking of clones during time course experiments (77, 78). The unique feature of Vidjil is that it also provides a web application linked to a patient database where one can keep records of all patients alongside their Ig-Seq data (77). The web application can visualize the data processed made by the Vidjil algorithm, or by other V(D)J analysis pipelines (61).

##### V(D)J Germline assignment and Clonal Grouping/Clonotyping

The platform uses as default a seed heuristic algorithm to perform the alignment and clustering of the clonotypes. This process is fast as no alignment is performed with germline database sequences in the first phase (62). A key feature provided by Vidjil is that it also supports data processed by other clonal grouping tools, such as IMGT-HighV-QUEST, IgBlast, MiXCR, IMSEQ, among others.

##### Diversity

Provides the Shannon-Wiener and Inverse Simpson Diversity indexes.

### Specialized Pipelines

The computational tools that perform clonal grouping/clonotyping and at least one feature for repertoire characterization are SONAR, TRigS, IMEX, Vidjil, IRProfiler, and VDJtools. Those whose main features focus solely on the repertoire characterization are DIVE, BASELINe, and AbSim.

#### SONAR

The Software for the Ontogenic aNalysis of Antibody Repertoires (SONAR) was specifically designed for analyzing the development of antibody lineages across time (58).

##### Pre-processing

The tool supports both single- and pair end-reads. It performs quality control and annotation on Ig-Seq data.

##### Clonal Grouping/Clonotyping

SONAR first clusters the reads based on assigned V and J genes. The transcripts in each group are then clustered based on their CDR3 nucleotide identity. Therefore, a clonal group contains all IG reads that share a common ancestor. The tool provides the option for seeded or unseeded lineage assignment.

##### Diversity

It provides VJ usage and CDR length distribution plots.

##### Evolution

SONAR is capable of tracking the development of specific antibody lineages across time. The visualization of the evolution is portrayed as longitudinal phylogenetic “birthday” trees. In the case of longitudinal phylogenetic birthday trees, SONAR identifies the sequences that appear at multiple time points and assigns a birthday based on the first observation of the read (58).

#### TRigS

##### Clonal Grouping/Clonotyping

By using the built-in tool *ClusterSeqs*, TRigS groups reads by their common ancestor based on single linkage clustering (79). Clonotyping of CDR3s can be defined at the nucleotide or amino acid levels by an identity threshold (Hamming distance divided by the read length). Furthermore, the output from the clonotyping can be easily graphed and translated into annotated lineage trees, showing the positions at which nucleotide/amino acid substitutions occur.

##### Diversity

The built-in tool “PlotGermline” creates histograms to display germline usage. It is also capable of plotting the relative usage of a requested germline gene. In the case of diversity indexes, TRigS is able to calculate the Gini index for each cluster created.

#### IMEX

##### Clonal Grouping/Clonotyping

In ImmuneExplorer (IMEX), the calculation of clonality can be defined by the user by choosing the amino acid or the nucleotide sequence or the V-(D)-J rearranged regions (80). The software enables the calculation of clonality based on the three CDRs (CDR1–3). Total numbers and relative frequencies of the clonotypes are given in tabular view.

##### Diversity

IMEX calculates sequence diversity using a more elaborated data mining approach based on the CDR3 (80). Furthermore, the software provides several different graphical representations to visualize the total gene and allele frequencies such as frequency histograms, heat maps, or bubble charts.

##### Convergence

It is capable of obtaining a list of unique CDR3 clonotypes for a data sample and searching for them in another sample. It also contains a visualization and tabular view to compare overlapping multiple data samples according to CDR3.

#### IRProfiler

Immune Repertoire Profiler (IRProfiler) runs in the Galaxy environment and delivers a variety of core immune repertoire quantification and comparison functionalities on high-throughput BCR sequencing data (81). IRProfiler receives annotated IMGT HIGH-V Quest reads, and other annotated high-throughput dataset, and incorporates the same fields as in the Summary Report provided by IMGT.

##### V(D)J Germline assignment

IRProfiler provides 11 different quality-filtering criteria —profiled within the *Data Filtering* function, which includes the removal of out-of-frame junctions, functionality of the V gene, unproductive reads, among others, that can be performed after VJ alignment.

##### Clonal Grouping/Clonotyping

The pipeline contains five different definitions of clonotype to suit different analysis purposes. All clonotype definitions are in terms of amino acid sequences. IRProfiler starts with the IMGT definition of clonotype, which includes the same V, D and J gene and the same amino acid sequence at the CDR3, and gradually transitions toward a less detailed definition.

##### Diversity

IRProfiler provides a summary of the clonotype quantification. This includes the dominant clonotype and its frequency, the total number of clonotypes and the total number of expanding clonotypes. Furthermore, it is capable of performing V and J gene usage.

##### Convergence

The *Public clonotypes* tool implemented by IRProfiler allows the identification of clonotypes present in more than one repertoire. For this IRProfiler receives the list of clonotypes for each repertoire and outputs a file containing the shared clonotypes accompanied by their frequencies in each input repertoire and repertoire counts. Moreover, multiple V or J gene repertoires can be compared with respect to the gene usages.

#### VDJtools

VDJtools is an open source framework which computes a wide set of statistics and is able to perform various forms of cross-sample analysis (35). VDJtools provides both tabular output and publication-ready plots.

##### Pre-processing

VDJtools takes as input already aligned and clonotyped Ig-Seq data. Besides supporting data processed with MiGEC, IgBlast, IMGT HIGH-V QUEST, Vidjil, MiXCR, or IMSEQ, the framework also provides further pre-processing key features; for example the *correct* tool, which is a built-in tool that performs frequency-based correction to eliminate erroneous clonotypes. The framework provides a variety of other filtering options, like filtering non-functional clonotypes, filtering out all clonotypes found in another sample, filtering by frequency, and filtering V(D)J segments that match a specified segment set.

##### Diversity

For each sample, VDJtools calculates basic statistics of read counts, mean clonotype size, and number of non-functional clonotypes. It determines VJ gene usage and spectra-typing (which refers to the distribution of clonotype abundance by CDR3 sequence length). As for diversity indexes, the framework provides a wide arrange of options such as Chao 1 or Efron-Thisted estimate for lower bound diversity and Shannon-Wiener index, Normalized Shannon-Wiener index and Inverse Simpson index for diversity.

##### Evolution

VDJtools performs an *all-*vs.*-all* intersection between an ordered list of samples for clonotype tracking. Results are visualized as clonotype tracking stack plots or heatmaps.

##### Convergence

The framework performs a comprehensive analysis of clonotype sharing for a pair of samples. Data can be visualized as scatter plots of overlapping clonotype abundance, abundance plots, hierarchical clustering dendrograms and pairwise overlap circos plots. A unique feature provided by VDJtools is the built in tool *CalcPairwiseDistances*, which performs an all-vs.-all pairwise overlap for a list of samples and computes a set of repertoire similarity measures.

#### DivE

##### Diversity

DivE is an R package specifically designed to estimate the total diversity of a sample through rarefaction curves (51). DivE fits various mathematical models to multiple nested subsamples of individual-based rarefaction curves and choses the best performing model to create the final rarefaction curve and diversity estimation (50, 51). The result is a new species richness estimator referred to as the *DivE estimator*. This new method was compared with some of the most widely used non-parametric total diversity estimators [Chao 1, abundance-based coverage estimator (ACE), Bootstrap and Good-Turing estimator] and resulted to be more accurate (24, 49–51).

#### BASELINe

##### Mutation Analysis

The statistical framework for Bayesian estimation of Antigen-driven SELectIoN (BASELINe) specializes in the analysis of somatic mutation patterns (53).The tool identifies the type (silent or point) and location (FWR or CDR) of the mutations, it calculates the Bayesian estimation of replacement frequency for every read and positive and negative selection pressure. Moreover, the tool is capable of performing a comparative analysis between groups of sequences derived from different germline V(D)J segments.

#### AbSim

AbSim is an R package designed to create simulations of antibody repertoires (5). It allows the user to control biologically relevant parameters such as total time for evolution, rate and method of SHM, number, and rate of V(D)J recombination events, baseline mutation rate, rate at which new sequences are produced, clonal frequency and V(D)J germline gene distribution (5). AbSim is the first repertoire simulation framework that enables the comparison of commonly used phylogenetic methods with regard to their accuracy in inferring antibody evolution.

### V(D)J Alignment

Since the V(D)J alignment is one of the most important and intricate steps in the Ig-Seq data processing, the following are stand-alone tools that focus solely on this computational task.

#### IgBlast

It derived from the commonly used BLAST algorithm to perform specialized Ig-Seq alignment and similarity searching (68). It is a web based application or it can be used as a command line tool that aligns Ig reads to the germline reference database of choice. Moreover, it allows to visualize what matches to the germline alleles, the details at the rearranged junctions, and the framework and CDR regions, which positioned IgBlast is a gold standard for V(D)J mapping. However, the output is not straightforward to parse and summarize to a readable clonotype abundance table containing CDR3 sequences, segment assignments and list of somatic hypermutations. This motivated the development of MIGMAP (35); a tool from VDJtools that wraps IgBlast and is designed to facilitate analysis immune receptor libraries profiled using high-throughput sequencing by IgBlast (49). MIGMAP extends IgBlast capabilities like assembling of clonotypes, application of various filtering options such as quality filtering for CDR3 N-regions and mutations, among others.

#### IMGT High V-Quest

The international ImMunoGeneTics information system (IMGT) is the most complete and used database for germline immune alleles (65). High V-QUEST is a web based standalone tool that allows the alignment of Ig reads to its germline allele database. It can handle up to 5 × 10^5^ reads simultaneously; larger files can be split using pRESTO. High V-QUEST identifies the closest V, D, and J alleles based on a global pairwise alignment of each read with different subsets of the IMGT reference database, followed by an evaluation by similarity. The standard output contains the reads with its respective closest V, D, and J allele, and its corresponding identity percentage/score. It also provides the FR and CDR delineations and the three CDR lengths. Furthermore, the *synthesis view* facilitates the visual comparison of sequences that express the same V gene and allele but differ in mutation locations and junctions.

#### HTJoinSolver

It is an application that introduced a dynamic programming approach method that uses conserved immunoglobulin gene motifs to improve the performance of aligning V(D)J segments (82). In order to run, one has to download the database from IMGT (or preferred database) to then upload it to the application. HTJoinSolver provides methods to download and re-format germline genes from IMGT. The results produced by the HTJoinSolver are the V, D, and J germline alleles that best align with the reads.

#### IMPre

The Immune Germline Prediction, IMPre, is a stand-alone tool designed to predict germline V/J genes and alleles derived from BCR repertoire data (83). IMPre mimics the reverse process of VDJ rearrangement and supports the discovery of new alleles. IMPre can process the rearranged sequences with or without the C region. When including the C region, it will be identified using previously reported C sequences. A simple fasta file containing the known germline database must be provided for IMPre to work correctly.

#### IgDiscover

IgDiscover is a stand-alone tool that supports the discovery of new alleles in heavy chains (VH), light kappa chains (VK) and light lambda chains (VL) genes (84). It can receive input data from pair end reads in FASTQ format or single end reads in either FASTQ or FASTA format. It requires a starting database of VH, VK or VL genes that are used for primary assignment —done by using IgBLAST. The output of IgDiscover is an individualized germline database that includes a dendrogram of the V, D, and J sequences, the V(D)J assignments and their expression counts. It was proven to identify germline V genes with 100% accuracy.

#### IGoR

The Inference and Generation Of Repertoires, IGoR, is a comprehensive tool that takes the repertoire reads and quantitatively characterizes the statistics of receptor generation (6). IGoR explores all possible recombination scenarios for the read and provides the probabilities of each; a robust feature that in some instances may outperform other pipelines. IGoR can be used to infer recombination models, to evaluate sequence statistics, and to create synthetic sequences using an already generated recombination model.

## Navigating Within the Pipeline Repertoire

Finally, we provide two simple analytical situations that exemplify on how the Supplementary Table 1 may serve to spot rapidly how the pipelines differ from one another, and at the same time, the alternatives that exists to carry out a specific computational task within the tools discussed in this work; for a practical representation of a thorough workflow, please refer to Figure 1 within the work by Chaudhary and Wesemann (21).

First, consider a study that requires identifying synonymous and non-synonymous mutations within the datasets. From the Mutation Analysis section in Supplementary Table 1, IgRec, ImmunediveRsity and BASELINe, provide this feature. On the contrary, if what is required from the mutation analysis is to understand how the samples are affected by positive or negative selection, the only option available from the table is BASELINe; either directly, or through the Immcantation Framework. In this second example consider that a large number of samples were multiplexed and a de-multiplexing tool would ease the pre-processing of data; pRESTO (Immcantation Framework) and MiGEC, might help on this task.

Since not all tools account for a data pre-processing feature —a step required prior to any type of analysis discussed throughout this review— in order to correct for amplification errors and filter by data quality, it is possible to make use of some other tools as long as the input and output data formats are compatible among tools. MiXCR and pRESTO are among the most commonly used tools for pre-processing for their versatility and compatibility for downstream analysis. Indeed, the pre-processing step is linked in many cases to the experimental design and library construction, and therefore can only follow specific pathways in confined workflows (i.e., sequencing platform, UMI incorporation, single-end, or paired-ends). For more thorough information on how a sequencing platform and library preparation may have an impact in selecting the pipeline for the pre-processing, the reader may refer to the work by Chaudhary and Wesemann (21) and Yaari and Kleinstein (23).

We notice that not all computational tools perform all types of analysis; and for those which overlap apparently, the focus may be on a different feature or statistical rationale. For this reason the Supplementary Table 1 is portrayed so that its printouts can be revisited frequently and help to easily spot the pipelines features and differences and more examples like these —that may include the amplification bias without UMI, or specific definitions of clonotype, diversity indexes, or even to identify what other tools perform identical type of analysis (in cases where results are difficult to interpret and may need an independent confirmation by a different software)—may be chosen straightforward.

## Perspectives

The current repertoire of computational pipelines offers an unprecedented opportunity to study the immune response in individuals and populations. The review presented here compiled the most widely used pipelines and the features by which they converge and diverge. The table accompanying this review aims to aid on when and why to use a specific pipeline, as well as helping to visualize the best experimental strategy in order to characterize the immune repertoires. As these computational tools evolve and new ones emerge, we expect this information may increase or modify as the pipelines adapt to new discoveries and technologic development. For this reason, we include the table provided in this work in a spreadsheet format so that the user can add, remove, or edit the information contained in it as the pipeline repertoire and features progress in the future. This file may be downloaded at the Flores-Jasso lab's repository. With the advent of high-throughput sequencing it is now possible to understand the dynamics of the immune response diversity and function. Novel techniques that allow sequencing the transcriptome landscape of single cells will also offer a more precise perspective of the metabolic changes associated to antibody production by B cells (85). Also single cell sequencing offers the advantage of determining the genes and transcripts that give rise to both, heavy and light chains of each antibody; making possible to determine more precisely the genetic relationships and dynamics of antibody repertoires (15, 59, 85). Yet another aspect of the antibody repertoire analysis that we envision will have an impact in the future of precision medicine is the extent at which immune repertoires overlap among individuals and populations (44). As many more studies are available, the robustness for the convergence analysis will allow framing a less blurred picture of our response to foreign epitopes as species. Importantly, with the aid of convergence analysis, the treatment and prevention of autoimmune diseases will become more evident; as the understanding of clonal dynamics of auto-antibodies will permit to better interpret the causes that trigger the immune system to recognize self-epitopes as foreign. It is our hope that this review help to navigate the current pipeline repertoire of computational tools with more ease and probe to be useful in attracting more research groups into this exciting area.

## Author Contributions

LL-S-J assembled Supplementary Table 1. LL-S-J, SEA-V, and CFF-J discussed and wrote the manuscript.

### Conflict of Interest Statement

The authors declare that the research was conducted in the absence of any commercial or financial relationships that could be construed as a potential conflict of interest.

## Supplementary Material

Supplementary Table 1 The Pipeline repertoire. The most common features for each pipeline are written into each square. Check symbols, a feature performed by the corresponding tool but there is scarce, or no detailed information about how the process is performed; empty squares, the corresponding feature is not included in the specific tool at the moment of assembling the table. The table can be downloaded in a spreadsheet format at https://github.com/Flores-JassoLab.

## Glossary of Terms

**Unique Molecular Identifiers (UMI, also referred to as UID)** are fully —or partially, randomly generated oligomers attached to DNA and RNA molecules in the first step of sequencing library preparation—before PCR amplification, that work as a identity tags for each input molecule (86–88). UMI make possible to detect contamination and to correct any PCR amplification bias (28, 89).

**Molecular Amplification Fingerprinting (MAF)** is a methodological process that consists on a stepwise incorporation of UMI/UID (28). It begins with single molecule tagging of first-strand cDNA during reverse transcription with a reverse-UID (RID) (which provides a unique tag to each transcript), to then continue by tagging each DNA-RID molecule during multiplex PCR amplification with a forward-UID (FID). Over-amplified molecules receive more FIDs than under-amplified ones. MAF allows implementing the normalization for multiplex amplification bias effects.

**Phred quality score (Also called Q score)** measures the quality of each nucleotide identified per read by high-throughput sequencing; a score is assigned to each nucleotide base-call (90). A Q score of 30, for instance, is equivalent to having 1 incorrect base call out of 1,000.

**Paired-ends reads** are the two sequence fragments obtained by high-throughput sequencing where the two ends of the same DNA molecule are sequenced. Depending on the initial fragment size and read length, the fragments can either overlap or not.

**Clonotype clustering** is a method to group into families the clonotypes observed derived from the same ancestor inferred at the nucleotide level (90). It helps to prevent the skewing of data by the over-representation of clones.

**Hierarchical clustering** is a method that seeks to construct a hierarchy of clusters. In Ig-Seq analysis, this method is commonly employed to cluster the clones in the repertoire dataset in order to identify the sequences that share a common ancestor and subsequently infer a clonal grouping (90, 91).

**Hamming distance** is the absolute count of the different sequences either at amino acid or nucleotide level between two sequences. It measures the minimum number of letter substitutions required to convert one sequence into the other.

**k-mer** refers to all the possible sub-sequences (of length *k*) from a read obtained through DNA Sequencing. For example, a dinucleotide is a k-mer, where *k* = 2.

**Multiplex**. In Ig-Seq experiments, it refers to simultaneously measure multiple samples in a single sequencing run by adding multiple primer pairs of known sequences in a PCR reaction mixture (28, 92).

**RACE**. The Rapid Amplification of cDNA Ends, RACE, is a method to characterize the 5′ and 3′ ends of a cDNA sequence (93). In 5′–RACE, for instance, the goal is to characterize the 5′ portion of an mRNA; cDNA synthesis is typically followed by 2 × 300 paired-ends sequencing.
